# Ambulatory care sensitive conditions hospitalization for emergencies rates in Colombia

**DOI:** 10.11606/S1518-8787.2019053000563

**Published:** 2019-04-26

**Authors:** Abel E González-Vélez, Claudia Carolina Colmenares Mejía, Eduardo Low Padilla, Sandra Yadira Moreno Marín, Paola Andrea Rengifo Bobadilla, Juan Pablo Rueda Sánchez, Mario Arturo Isaza Ruget

**Affiliations:** I Keralty. Vicepresidencia Global de Salud. Departamento de Innovación. Bogotá, Colombia; II Fundación Universitaria Sanitas. Centro de Investigaciones en Ciencias de la Salud. Bogotá, Colombia; III Keralty. Vicepresidencia Global de Salud. Gerencia de Excelencia Clínica. Bogotá, Colombia; IV Keralty. Vicepresidencia Global de Salud. Bogotá, Colombia; V Entidad Promotora de Salud Sanitas. Vicepresidencia de Salud. Bogotá, Colombia; VI Entidad Promotora de Salud Sanitas. Presidencia de Salud. Bogotá, Colombia; VII Laboratorio Clínica Colsanitas. Presidencia Ejecutiva de Laboratorio. Bogotá, Colombia

**Keywords:** Patient Admission, Emergency Identification, Hospitalization, Emergency Medical Services

## Abstract

**OBJECTIVE:**

To analyze the emergency hospitalizations trend for ambulatory care sensitive conditions between 2011 and 2015 in a health insureance company of the Colombian Social Security General System.

**METHODS:**

A log-linear analysis based on age-adjusted hospitalization rates for ambulatory care sensitive conditions in the *Entidad Promotora de Salud Sanitas* was used to estimate the annual percentage change in these rates and to identify joinponts of the rates. Data was collected from administrative sources.

**RESULTS:**

There were 38,530 hospitalizations for ambulatory care sensitive conditions in 26,501 *Entidad Promotora de Salud Sanitas* enrollees, with a significant decrease in hospitalization rates. The annual percentage change estimated for the period was -9.5% with no significant joinpoints throughout the time interval.

**CONCLUSIONS:**

A significant reduction in hospital admissions due to ambulatory care sensitive conditions in Entidad Promotora de Salud *Sanitas* enrollees were reported for the last five years in this study.

## INTRODUCTION

In order to promote equity and cost effectiveness in the provision of health services, Colombia adopted in 2011 the primary health care strategy as part of the health system reform ^1^ . In this reform, the Colombian state transfers ex-ante management of health risks to insurance companies, beyond the contingency of the disease ^2^ .

The *Entidad Promotora de Salud* (EPS) *Sanitas* implemented a care model based on primary care units (PCU), which has a gatekeeping function and where activities of induced demand for maintenance of health and identification of specific risks are developed. Thus, programs for management of diseases such as hypertension, diabetes, chronic obstructive pulmonary disease and other conditions are implemented in the PCU, through which EPS carries out prevention, diagnosis, treatment, rehabilitation and follow-up in the insured population ^3^ .

The evaluation of the primary health care strategies of several health systems around the world have been studied. Among the methodologies used, the analysis of ambulatory care sensitive conditions (ACSC) has been regarded as an indicator of primary care performance. This indicator has been used as a proxy measure for potentially preventable admission due to acute and chronic diseases^4–6^. High hospitalization rates for different ACSC may indicate poor quality of care at the primary care level^7–9^. This evaluation also allows to identify individuals for targeting of interventions to reduce preventable admissions ^10^ ^,^ ^11^ .

In Colombia, the information related to ACSC hospitalizaitions is limited. Therefore, this study aims to determine the trend of the hospitalization incidence by these conditions among enrollees of a health insurer from the Colombian Social Security General System (SSGS).

## METHODS

A study on the incidence of hospitalizations by ACSC was carried out analyzing a retrospective cohort study by EPS *Sanitas* enrollees between 2011 and 2015. During this period, this EPS covered 8.5% of the total insured population covered by the contributory regime of the SSGS. This population was located in regions such as Bogotá, Barranquilla, Bucaramanga, Cali, Medellín, and Central East Region. Economically active EPS *Sanitas* enrollees and their families belong to the contributory regime. Hospitalizations were identified based on the medical record performed by the Audit Department of this EPS. This record includes information generated by the hospital network of EPS *Sanitas* . During hospitalization, patients are followed daily or every other day by the Audit Department, which registers variables such as age, sex, type of admission (elective *versus* emergency), principal diagnosis, length of stay, and discharge status (alive *versus* dead). The hospitalizations selected corresponded to emergency admissions whose main causes of admission were acute ACSC (urinary infection, cellulitis, gangrene, pelvic inflammatory disease, dehydration, malnutrition, gastric ulcer, gastroenteritis, ear, nose and throat infections) or chronic ACSC (Chronic Obstructive Pulmonary Disease (COPD), asthma, Congestive Heart Failure (CHF), diabetes, epilepsy, high blood pressure and anemia), proposed by Bardsley et al. ^7^ Additional to the original ICD 10 codes list, other codes related to the ACSC were added, since they were identified as commonly used in the diagnostic classification process in our context ( Box ). All readmissions, occurring within 30 days after a previous hospitalization, were excluded from this analysis. These admissions were considered not related to healthcare quality delivered in primary care.

**Box:** Ambulatory Care Sensitive Conditions list of codes ICD-10.

Condition	ICD-10
Chronic ACSC	
Anginas	I20; I200; I201; I208; I209; I24; I240; I248; I249; I25; I251; I255; I256; I258; I259
Asthma	J45; J450; J451; J458; J459; J46
COPD	J20; J200; J201; J202; J203; J204; J205; J206; J207; J208; J209; J41; J410; J411; J418; J42; J43; J430; J431; J432; J438; J439; J44; J440; J441; J448; J449; J47
Congestive heart failure	I110; I50; I500; I501; I509; J81
Seizures and epilepsy	G40; G400; G401; G402; G403; G404; G405; G406; G407; G408; G409; G41; G410; G411; G412; G418; G419; O15; O150; O151; O152; O159; R56; R560; R568
Complications of diabetes	E10; E100; E101; E102; E103; E104; E105; E106; E107; E108; E11; E110; E111; E112; E113; E114; E115; E116; E117; E118; E12; E120; E121; E122; E123; E124; E125; E126; E127; E128; E13; E130; E131; E132; E133; E134; E135; E136; E137; E138; E14; E140; E141; E142; E143; E144; E145; E146; E147; E148
Hypertension	I10 ; I119
Iron deficiency anemia	D50; D501; D508; D509
Acute ACSC	
Cellulitis and gangrene	L03; L030; L031; L032; L033; L038; L039; L04; L040; L041; L042; L043; L048; L049; L08; L080; L081; L088; L089; L88; L980; L983; R02
Dehydration	E86; E87; E878; F505; K910; P741; P920; R11
Dental conditions	A690; B084; B370; K02; K021; K029; K03; K033; K038; K04; K046; K047; K05; K050; K06; K068; K08; K088; K098; K099; K12; K120; K121; K122; K13
Ear, nose and throat infections	H66; H660; H661; H662; H663; H664; H669; H67; H670; H671; H678; J02; J020; J028; J029; J03; J030; J038; J039; J06; J060; J068; J069; J312; J39
Gastroenteritis	K52; K521; K522; K528; K529
Nutritional deficiencies	E40; E41; E42; E43; E44; E440; E441; E45; E46; E55; E550; E64; E640; E643
Pelvic inflammatory disease	N70; N700; N701; N709; N73; N730; N731; N732; N733; N734; N735; N736; N738; N739; N74; N740; N741; N742; N743; N744; N748
Perforated or bleeding ulcer	K250; K251; K252; K254; K255; K256; K260; K261; K262; K264; K265; K266; K270; K271; K272; K274; K275; K276; K280; K281; K282; K284; K285; K286
Urinary tract infection or pyelonephritis	N10; N11; N110; N111; N118; N119; N12; N136; N30; N300; N301; N302; N303; N308; N309; N390

ACSC: ambulatory care sensitive conditions; COPD: pulmonary disease, chronic obstructive

### Statistical Analysis

A descriptive analysis of the data was performed. Quantitative variables were summarized using measures of central tendency and dispersion, while qualitative variables were described by absolute and relative frequencies. To determine the frequencies of hospitalization, the rates were calculated as the proportion (cumulative incidence) of hospital discharges for EPS *Sanitas* enrollees by year. Rates were stratified by age groups, and by type of ACSC (acute *versus* chronic). In order to analyze the trend of hospitalizations, a log-linear model was used based on age-adjusted rates according to the type of ACSC, and for each of the conditions in Box . This method also allowed to estimate the annual percentage change (APC) of these rates. The standard population used in the standardization of age rates was the Colombian census population of 2005. The rates were adjusted by age, since during the study period the composition of the insured population of EPS *Sanitas* changed by age. It included younger people in the most recent years analyzed. There were not changes in sex distribution during the study period, so the analysis adjusted by sex was similar to the one presented. The descriptive analysis was carried out using Stata 13. Trend analysis was conducted using Joinpoint Regression Program 4.3.10. The p-value of the statistical significance used for the whole analysis was ≤ 0.05.

## RESULTS

Between January 2011 and December 2015, there were 38,530 episodes of hospitalization by ACSC in 26,501 patients of EPS *Sanitas* . Of these, 58.7% were women and the median age was 48 years (p25-p75: 12–71 years). In 453 (1.7%) cases, the final discharge status was due to patient death. As for hospital stay, the median was four days (p25-p75: 3–6 days), with the highest admission rate in the regional Bogotá (61%). Sociodemographic characteristics according to the type of ACSC (acute *versus* chronic) are shown in Table 1 .

**Table 1:** Sociodemographic characteristics by type of ACSC.

Characteristic	Acute ACSC	Chronic ACSC
Patients – n	14,873	11,628
Age – median (p25–p75)	32 (7–62)	62 (30–76)
Sex – n (%)		
Male	5,089 (34.2%)	5,857 (50.4%)
Female	9,784 (65.8%)	5,771 (49.6%)
Deaths – n (%)	92 (0.6%)	361 (3.1%)
Hospitalizations – n	19,256	19,274
Regional – n (%)		
Bogotá	11,568 (60.1%)	11,926 (61.9%)
Barranquilla	1,954 (10.2%)	1,956 (10.2%)
Medellín	1,655 (8.6%)	2,249 (11.7%)
Cali	1,196 (6.2%)	1,415 (7.3%)
Bucaramanga	1,423 (7.4%)	922 (4.8%)
Central east	1,460 (7.6%)	806 (4.2%)
Hospital stay – median (p25–p75)	4 (3–6)	4 (3–7)
Year – n		
2011	3,472	4,362
2012	3,880	3,808
2013	4,057	3,590
2014	3,807	3,710
2015	4,040	3,804

ACSC: ambulatory care sensitive conditions

The ACSC hospitalizations crude rate decreased from 112.4 hospitalizations per 10,000 enrollees in 2011 to 71.7 hospitalizations per 10,000 enrollees in 2015. Age-adjusted ACSC hospitalization rates changed from 102.1 hospitalizations per 10,000 enrollees in 2011 to 68.4 hospitalizations per 10,000 enrollees in 2015. On the other hand, acute ACSC crude rates ranged from 49.8 (2011) to 36.9 (2015) hospitalizations per 10,000 enrollees, while rates for chronic conditions were between 62.6 (2011) and 34.8 (2015) hospitalizations per 10,000 enrollees. Acute and chronic age-adjusted ACSC rates are presented in Table 2 .

**Table 2:** Age-adjusted ACSC hospitalization rates (x 10,000 enrollees).

ACSC	2011		2012		2013		2014		2015		PCA (%)
				
	Hospitalizations	Rate (95%CI)	Hospitalizations	Rate (95%CI)	Hospitalizations	Rate (95%CI)	Hospitalizations	Rate (95%CI)	Hospitalizations	Rate (95%CI)	
Acute	3,472	49.55 (47.75–51.34)	3,880	51.81 (50.02–53.60)	4,057	49.59 (47.93–51.24)	3,807	39.50 (38.14–40.85)	4,040	37.58 (36.33–38.82)	-8.1
Urinary tract infections or pyelonephritis	2,222	31.17 (29.75–32.59)	2,381	30.97 (29.59–32.34)	2,538	30.48 (29.19–31.78)	2,320	23.39 (22.36–24.42)	2,558	23.10 (22.14–24.06)	-8.5
Cellulitis gangrene	827	11.74 (10.88–12.61)	931	12.25(11.39–13.10)	919	11.13 (10.35–11.91)	886	9.04 (8.40–9.68)	856	7.97 (7.39–8.54)	-10.4
Pelvic inflammatory disease	157	2.07 (1.74–2.39)	181	2.28 (1.95–2.62)	202	2.25 (1.93–2.56)	186	1.73 (1.48–1.98)	196	1.60 (1.37–1.82)	-8.0^a^
Dehydration	74	1.38 (1.05–1.72)	128	2.23 (1.82–2.65)	126	1.93 (1.57–2.29)	146	2.06 (1.71–2.41)	132	1.53 (1.25–1.80)	-1.5^a^
Ear, nose, and throat infections	71	1.43 (1.09–1.78)	105	1.99 (1.59–2.38)	120	2.01 (1.64–2.39)	105	1.6 (1.29–1.91)	136	1.86 (1.54–2.18)	1.9^a^
Bleeding or perforated ulcer	20	0.20 (0.11–0.28)	53	0.49 (0.35–0.63)	61	0.51 (0.38–0.64)	75	0.56 (0.43–0.69)	57	0.38 (0.28–0.48)	6.2^a^
Dental conditions	44	0.83 (0.58–1.08)	58	1.05 (0.77–1.33)	53	0.81 (0.58–1.04)	51	0.71 (0.51–0.92)	52	0.64 (0.46–0.82)	-9.2^a^
Gastroenteritis	48	0.58 (0.41–0.75)	32	0.36 (0.22–0.50)	29	0.35 (0.21–0.48)	29	0.30 (0.18–0.41)	43	0.41 (0.28–0.54)	-8.8^a^
Nutritional deficiencies	9	0.14 (0.04–0.24)	11	0.19 (0.06–0.31)	9	0.12 (0.03–0.21)	9	0.10 (0.03–0.18)	10	0.11 (0.04–0.18)	-11.2^a^
Chronic	4,362	49.95 (48.31–51.58)	3,808	41.38 (39.92–42.84)	3,590	38.04 (36.66–39.42)	3,710	32.89 (31.73–34.05)	3,804	30.79 (29.73–31.85)	-11.3
Anginas	1,040	9.62 (9.03–10.21)	790	6.76 (6.28–7.24)	677	5.38 (4.97–5.79)	808	5.73 (5.33–6.13)	867	5.64 (5.26–6.02)	-24.8^a^ [2011–2013]^b^ 3.6^a^ [2013–2015]^b^
COPD	993	8.43 (7.9–8.96)	913	7.13 (6.66–7.59)	740	5.45 (5.06–5.85)	789	5.21 (4.85–5.58)	733	4.47 (4.15–4.80)	-18.7^a^ [2011–2013]^b^ -10.2^a^ [2013–2015]^b^
Asthma	713	14.49 (13.40–15.58)	626	12.29 (11.30–13.27)	710	12.96 (11.99–13.93)	584	8.97 (8.22–9.71)	580	8.13 (7.46–8.80)	-13.5
CHF	625	5.34 (4.92–5.77)	580	4.61 (4.23–4.99)	582	4.37 (4.00–4.73)	678	4.61 (4.26–4.96)	707	4.38 (4.05–4.70)	-3.9^a^
Diabetes	391	4.08 (3.66–4.51)	367	3.71 (3.30–4.11)	349	3.24 (2.88–3.60)	316	2.55 (2.25–2.84)	355	2.55 (2.28–2.83)	-12.2
Epilepsies	307	5.07 (4.46–5.67)	314	4.85 (4.28–5.43)	340	4.92 (4.36–5.47)	385	4.64 (4.15–5.13)	429	4.69 (4.22–5.16)	-1.9
Hypertension	273	2.66 (2.33–2.98)	184	1.63 (1.39–1.88)	155	1.31 (1.10–1.52)	113	0.84 (0.68–1.00)	113	0.78 (0.63–0.93)	-27.6
Anemias	20	0.25 (0.13–0.38)	34	0.40 (0.25–0.56)	37	0.42 (0.27–0.56)	37	0.35 (0.23–0.47)	20	0.16 (0.08–0.23)	31.2^a^ [2011–2013]^b^ -38.5^a^ [2013–2015]^b^

ACSC: ambulatory care sensitive conditions; COPD: pulmonary disease, chronic obstructive; CHF: congestive heart Failure; APC: annual percentage change

^a^ Values for which the APC is not significantly different from 0 for an α = 0.05.

^b^ In those trends where there was a significant change point in the period, the bracket values ([ ]) indicate the time segment for which each PCA is provided.

The analysis of hospitalizations according to ACSC type and age groups showed that the rates were highest in children under one year of age, followed by those over 74 years when the rates were about acute conditions, while among chronic ACSC, rates were higher in older groups. In contrast, lower rates of hospitalization were seen in children older than five years and in young adults for both acute and chronic conditions ( Figure ).

**Figure:**
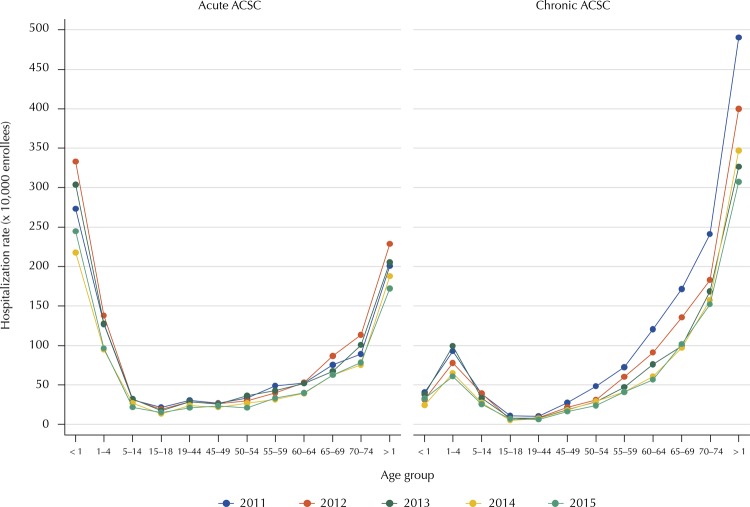
Hospitalization rates by age group according to ACSC type.

As for trend analysis, between 2011 and 2015 there was a significant decrease in age-adjusted ACSC hospitalization rates (APC = -9.5%) with no significant joinpoints throughout the time interval. Likewise, there was a downward and significant trend in adjusted hospitalization rates due to acute (APC = -8.1%) and chronic conditions (APC = -11.3%), with no significant joinpoints in any of these trends.

In order of frequency, angina, COPD, asthma, CHF and diabetes represented the top five causes of hospitalization for chronic conditions, accounting for 85% of these hospitalizations, whereas among acute conditions, the top two causes (urinary infections/pyelonephritis and cellulitis) had the same proportion of hospitalizations as the top five chronic conditions. Asthma, diabetes, epilepsy and hypertension, among chronic ASCC, as well as urinary infection/pyelonephritis and cellulitis/gangrene, among acute ASCC, showed negative and significant APC in age-adjusted hospitalization rates. The trend in hospitalization rate for hypertension had the highest magnitude of change (APC = -27.6%). The other conditions studied showed stable trends in hospitalization rates ( Table 2 ). Finally, only anginas, COPD and anemias had significant joinpoints of their trends in 2013.

## DISCUSSION

This study quantified the frequency of hospitalization for ACSC in the insurer’s enrollees of the Colombian SSGS during a five-year period. While younger patients were predominantly hospitalized for acute conditions, older adults were admitted for chronic causes. Although both acute and chronic ACSC hospitalizations presented downward trends, the latter did so in greater magnitude. In addition, more chronic conditions than acute had a significant decrease in the trend of hospitalization, in which only the chronic group had conditions with significant points of change in hospitalization rates between 2011 and 2015.

The differential susceptibility of the extremes of life according to the type of ACSC analyzed is a finding consistent with previous studies^12–14^. Thus, studies in Portugal and France have shown that chronic conditions such as COPD, heart failure and hypertension are responsible for increasing rates of hospitalization as age increases, especially in adults 60 years or older ^7^ ^,^ ^12^ ^,^ ^14^ . Similarly, conditions such as UTI and pyelonephritis, which together have been the most frequent cause of hospitalization for acute ACSC in this study, have shown peaks of incidence in both the younger and older age groups in this study as well as in previous studies ^12^ ^,^ ^13^ .

Trend evaluation of hospitalization for ACSC has shown variable results among countries, and even for the same country according to the published study^7,15–19^. While Rosano et al. ^15^ reported a significant decrease in the trend of these hospitalizations in Italy (CAP: -16.4%, 2001–2008), as Niti et al. ^16^ in Singapore did (CAP: -15.0%, 1991–1998), Bardsley et al. ^7^ published an analysis in which hospitalization rates for ACSC increased by 21% between 2001 and 2011 in England. Downward trends of smaller magnitude have been observed in England (CAP: -10%, 2002–2009), Spain (CAP: -4.2%, 2002–2013) and Brazil (CAP: -3.7%, 1998–2009)^17–19^. On the other hand, an absence in the trend of hospitalization for ACSC has been documented in Ireland, Portugal, Slovenia and Spain ^13^ ^,^ ^17^ .

According to the specific ACSC, this study like others already published, has shown a decrease in the trend of hospitalizations for conditions such as diabetes, asthma and COPD ^15^ ^,^ ^18^ ^,^ ^20^ . However, hypertension has shown variable results in the literature, some reports have found an increase in hospitalization rates for England, meanwhile in Brazil the rate remained stable ^7^ ^,^ ^19^ . Nonetheless, in this study, the hospitalization rates for hypertension have shown the highest decrease in the trend.

In our study, the observed decrease in the trend of hospitalization for ACSC, especially for chronic conditions, may be due to several factors, including the performance of *Entidad Promotora de Salud Sanitas* within the Colombian SSGS, the socioeconomic characteristics of its enrollees, as well as the strategy of primary care implemented in its PCU to strengthen the risk management of its users. In Colombia, previous analyses of the performance of SSGS’s insurers have shown that this EPS has presented higher scores when compared to other insurers of the contributory regime ^21^ ^,^ ^22^ . Among the interventions adopted by EPS at the level of primary care, there is more investment in technological and human resources in its PCU in recent years, better identification and follow-up of high-risk patients, as well as an integrated model of care based on primary care as a gatekeeper to health services ^3^ .

Within the limitations of this study, it is to be expected that diagnostic classification errors could have occurred during the medical coding process, without any evidence that might indicate this has occurred differently between the conditions analyzed or over time. Likewise, the comparability of our results in the local context was difficult because of the limited number of published studies of this type in Colombia, as well as differences in the populations chosen. In one of them, the authors analyzed hospitalizations by ACSC in only five public hospitals serving the population of the subsidized SSGS regime ^23^ .

On the other hand, the magnitude of the variability in the patients included according to age was not evaluated, therefore, it’s possible that biases related to the socioeconomic and sanitary profile of the study population were inserted. Additionally, only emergency admissions were used, excluding elective admissions, which may have generated data collection bias by classification.

In conclusion, to our knowledge the results of this study constitute the first analysis of hospitalization for ACSC performed by an insurer in Colombia. The rates obtained represent an indirect performance indicator of the *Sanitas* EPS in primary care, observing a significant reduction in hospital admissions for ACSC in the last years. At the same time, the observed rates will allow the decision-making process in EPS to be guided by actions and policies necessary to reduce hospitalizations for those ACSC that, by their frequency or trend, still constitute an opportunity to improve primary healthcare performance.
